# NRF2 Activation by Nitrogen Heterocycles: A Review

**DOI:** 10.3390/molecules28062751

**Published:** 2023-03-18

**Authors:** Melford C. Egbujor, Paolo Tucci, Ugomma C. Onyeije, Chigbundu N. Emeruwa, Luciano Saso

**Affiliations:** 1 Department of Chemical Sciences, Rhema University Nigeria, Aba 453115, Nigeria; 2 Department of Clinical and Experimental Medicine, University of Foggia, 71122 Foggia, Italy; 3 Department of Pure and Industrial Chemistry, Nnamdi Azikiwe University, Awka 420007, Nigeria; 4 Department of Physiology and Pharmacology, Vittorio Erspamer, Sapienza University of Rome, 00161 Rome, Italy

**Keywords:** nitrogen heterocycles, NRF2, HO-1, NQO1, antioxidant, anti-inflammatory, neurodegenerative diseases

## Abstract

Several nitrogen heterocyclic analogues have been applied to clinical practice, and about 75% of drugs approved by the FDA contain at least a heterocyclic moiety. Thus, nitrogen heterocycles are beneficial scaffolds that occupy a central position in the development of new drugs. The fact that certain nitrogen heterocyclic compounds significantly activate the NRF2/ARE signaling pathway and upregulate the expression of NRF2-dependent genes, especially HO-1 and NQO1, underscores the need to study the roles and pharmacological effects of *N*-based heterocyclic moieties in NRF2 activation. Furthermore, nitrogen heterocycles exhibit significant antioxidant and anti-inflammatory activities. NRF2-activating molecules have been of tremendous research interest in recent times due to their therapeutic roles in neuroinflammation and oxidative stress-mediated diseases. A comprehensive review of the NRF2-inducing activities of *N*-based heterocycles and their derivatives will broaden their therapeutic prospects in a wide range of diseases. Thus, the present review, as the first of its kind, provides an overview of the roles and effects of nitrogen heterocyclic moieties in the activation of the NRF2 signaling pathway underpinning their antioxidant and anti-inflammatory actions in several diseases, their pharmacological properties and structural–activity relationship are also discussed with the aim of making new discoveries that will stimulate innovative research in this area.

## 1. Introduction

Nitrogen-based heterocyclic compounds constitute an important class of heterocycles in drug discovery due to their vast medicinal applications. It is well established that nitrogen heterocyclic scaffolds are often present as common cores in a variety of pharmaceutical products. This implies that nitrogen heterocycles play essential roles in modern drug design and discovery. Currently, over 85% of all biologically active compounds are heterocycles or contain at least a heterocyclic moiety, and most frequently, nitrogen heterocycles function as the backbones of these complex structures [1]. The applications of nitrogen heterocycles in drug design and development have been reviewed by [2]. Many of them have been found to possess anticancer activities and good physicochemical properties [3]. Thus, the presence of an *N*-based heterocyclic moiety may improve the adsorption, distribution, metabolism and excretion (ADME) and toxicological properties of drug molecules.

Reactive oxygen species (ROS) and reactive nitrogen species (RNS) play essential physiological roles at moderate concentrations. However, when there is a disequilibrium between the rate of production of ROS and the rate at which antioxidant defenses neutralize them, oxidative stress occurs and results in oxidative damage and cell death [4,5]. Fortunately, some essential antioxidant molecules and detoxifying enzymes have been developed by cells as adequate defenses against oxidative stress. NRF2 protein is a notable antioxidant molecule that regulates cellular redox homeostasis; thus, its activation represents an effective antioxidant strategy against electrophilic and oxidative stress [6].

Consequently, NRF2-related pathways have become important therapeutic targets in drug discovery for inflammation and oxidative stress-mediated diseases [7,8]. The NRF2/KEAP1 signaling pathway modulates the antioxidant and cytoprotective responses of an organism to a great extent [6]. NRF2 is a transcription factor that is made up of about 605 amino acids and 7 functional domains known as Neh1-Neh7. The Neh1 domain is composed of a cap “n” collar basic region and the leucine zipper domain, which enables DNA binding and a nuclear localization signal that accounts for NRF2 nuclear translocation [9,10]. The Neh2 domain is responsible for NRF2 stability and its ubiquitination by KEAP1, while the Neh 3–5 domains facilitate NRF2 interaction with several coactivators [11,12,13,14]. The Neh6 domain binds to a β-transducin repeat-containing protein (β–TrCP), through which it enhances NRF2 ubiquitination, while the Neh7 domain enables NRF2 to bind to the retinoic X receptor and causes the inhibition of the NRF2–ARE pathway [15,16].

When the condition is physiologically normal, NRF2 is bound to KEAP1, its negative regulator in the cytosol. This promotes NRF2 ubiquitination by a cullin 3-based ubiquitin E3 ligase and NRF2 proteasomal degradation [17]. Under oxidative or electrophilic stress, KEAP1 cysteine residues are oxidized to cystine. This transformational process facilitates a conformational change in the protein that inhibits NRF2 ubiquitination and promotes the formation of the non-functional NRF2/KEAP1 complex, which does not allow for the release of NRF2. This enables the newly translated NRF2 to by-pass KEAP1 and undergo nuclear translocation; it binds to the antioxidant response element (ARE) sequence and facilitates the transcription of NRF2-dependent genes that codify the synthesis of antioxidant enzymes such as SOD, NQO1, HO-1, CAT, GCL, GPX and GR [18,19].

Comparatively, NRF2 and NF-κB (nuclear factor kappa-light-chain-enhancer of activated B cells) are both transcription factors that are crucial for the regulation of oxidative stress, inflammation, gene expression and other physiological processes. The cellular antioxidant defense and detoxification pathways are largely regulated by NRF2 [17,18,19]. NF-κB, on the other hand, is a transcription factor involved in immune and inflammatory responses, controlling genes related to cytokines, chemokines and adhesion molecules [20,21]. It is normally bound to its inhibitor IκB and moves to the nucleus, where it binds to κB sites in order to activate gene expression. While their functions are distinct, there is an interplay between these signaling pathways as their activities are usually inversely correlated. Research suggests that NRF2 can inhibit NF-κB activation by suppressing proinflammatory cytokines/chemokines production; while NF-κB can result in ROS generation, causing disruption of the KEAP1–NRF2 complex which can consequently lead to NRF2 activation [22,23,24]. However, the overall relationship between NRF2 and NF-κB is quite complex and context-dependent; hence, more studies are required to fully elucidate their functional effects in various physiological and pathological conditions. 

The systematic evaluation of the functional effects of nitrogen heterocyclic molecules through in vivo and in vitro studies gives insight into their pharmacological profile. This information will determine their suitability as new drug candidates and identify their therapeutic indications. Furthermore, it could be an essential tool for the development of newer derivatives of nitrogen heterocycles with better NRF2-mediated antioxidant and anti-inflammatory activities. The pharmacological profile of NRF2-activating nitrogen heterocyclic compounds will be further discussed in Section 3. Similarly, structure–activity relationships (SAR) analysis is employed as an essential tool in primary screening to lead optimization of drug discovery. It helps to minimize the cost of designing new potentially bioactive molecules with minimal side effects. A good knowledge of the SAR of nitrogen heterocyclic molecules will enable researchers to explore their existing bioactive moieties and equip them with the information required for structural modification and optimization of antioxidant, anti-inflammatory and NRF2-inducing activities. The SAR will be further discussed in Section 4.

The present review discusses the potential roles of natural and synthetic nitrogen-based heterocycles in the activation of the NRF2 signaling pathway, their pharmacological properties and their structure–activity relationships. Their NRF2-mediated neuroprotective and therapeutic effects in inflammation and oxidative stress-mediated diseases such as Parkinson’s disease, Alzheimer’s disease, Huntington’s diseases, cancer, and many more, are explored.

## 2. Nitrogen Heterocycles as Modulators of the NRF2 Pathway

The tendency of the nitrogen atom to readily form hydrogen bonding and various weak interactions with biological targets has distinguished N-based heterocyclic scaffolds as building blocks for a couple of drug candidates and expanded their utility in several therapeutic applications. The nitrogen atom of nitrogen heterocycle has a lone pair of electrons, which act as a hydrogen bond acceptor, resulting in the formation of a hydrogen bond (hydrogen atom bonded to an electronegative atom) network which enhances the stability of the nitrogen heterocycle and its interactions with diverse biological molecules [25,26,27]. Thus, both saturated and unsaturated N-based heterocycles are bioactive molecules of utmost medicinal importance. A large body of evidence has shown that N-based heterocycles and their analogues possess an interesting neuroprotective profile and exhibit significant induction of the NRF2–ARE antioxidant responses. Here, we categorize the NRF2-inducing activity of *N*-based heterocycles based on the size of the heterocyclic ring. Three-membered and four-membered nitrogen heterocyclic rings such as aziridines and azetidines, respectively, and their derivatives have been found to exhibit anti-oxidative and neuroprotective effects [28,29,30]. However, these compounds are yet to be explored for NRF2-inducing activity.

### 2.1. Five-Membered Nitrogen Heterocycles and NRF2 Activation

Five-membered heterocyclic rings are commonly found in pharmaceuticals. It can be stipulated that their chemical structures permit variable interactions with essential biomolecules, hence their predominance in pharmaceuticals. Five membered nitrogen heterocycles such as pyrrole, imidazoles, pyrazoles and many more are components of the best-selling heterocyclic pharmaceuticals [31]. Currently, the pyrrole derivative, 3-carboxylic acid pyrroles have been patented as active NRF2 regulators (US2020/0031820A1). Moreover, pyrrole-2-carbaldehydes exert neuroprotective effects against oxygen-glucose deprivation/reperfusion injury by modulating NRF2 and Nuclear Factor kappa B (NF-ĸB) in PC12 cells [32].

#### 2.1.1. Pyrrolidine/Pyrroline Analogues

Pyrrolidine and pyrroline are saturated and unsaturated five-membered *N*-heterocycles, respectively, with one nitrogen heteroatom. Pyrrolidine is naturally found in alkaloids and is also an essential constituent of natural and synthetic drugs. Pyrrolidine is conventionally synthesized by the reaction of 4-chlorobutan-I-amine with a strong base [33]. Its derivatives are also synthesized by electroreductive cyclization using imine and terminal dihyloalkanes [34]. Recent synthetic methods for pyrrolidines have been reviewed by [35]. A pyrrolidine derivative known as pyrrolidine dithiocarbamate (**1**) (Table 1) has been reported as a potent inducer of the NRF2 signaling pathway [36]. It inhibits oxidative stress, decreases lipid peroxidation, and exerts neuroprotection via the activation of NRF2 signaling pathway in astrocytes. Delen and co-workers [37] reported that in addition to reducing the expressions of NF-ĸB and Prokineticin 2 (PK2) levels, pyrrolidine dithiocarbamate (**I**) exerts a protective effect against methotrexate-induced testicular damage via upregulating the expression level of NRF2. Contrarily, it has been reported that while pyrrolidine dithiocarbamate deactivates NF-ĸB and upregulates some antioxidant enzymes, its administration has no effect on NRF2/KEAP pathway in dextran sodium sulfate (DSS)-induced colitis [38]. This could be a result of the fact that pyrrolidine dithiocarbamate (**1**) lacks the ability to alter the NRF2-inducing effect of DSS, which is also a potent NRF2 inducer. It is important to mention that compound **1** also induces the expression of the glutamate cysteine ligase modulatory gene in HepG2 cells. Although the nuclear localization of NRF2 has been implicated in this process, the activation of the extracellular regulated kinase (ErK) is required for full NRF2 activation. Treatment of HepG2 cells with compound **1** results in the release of NRF2 from KEAP1 and influences the expression level of GCL [39]. In an attempt to demonstrate that NRF2 modulates neurogenesis and exerts a protective effect against Aβ toxicity, neural progenitor cells (NPCs) were treated with compound **1,** and the growth of NPC neurospheres was observed and neuronal differentiation was increased by it via NRF2 activation [40]. Pyrroline derivative (**2**) (Table 1) exerts protection against oxidative stress and hyperphosphorylation in neurodegenerative diseases via the activation of the NRF2–ARE pathway and upregulation of the expression of protein levels of HO-1 and NQO1 [41].

#### 2.1.2. Pyrazoles

Pyrazoles are unsaturated five-membered N-heterocyclic rings containing two nitrogen atoms at adjacent positions. Owing to their myriad of pharmacological activities, they are one of the most prominent classes of compounds among the azole group. Thus, they are components of well-established drugs such as celecoxib, lenazole, rimonabant, and many more [42,43,44]. Pyrazoles are commonly prepared by reacting α,β-unsaturated aldehydes with hydrazine followed by dehydrogenation [45]. They are also synthesized by electrophilic cyclizations of α,β-alkynic hydrazones by iodine [46]. The various methods involved in the synthesis of pyrazoles have been reviewed by [47]. Pyrazoles exhibit significant anti-inflammatory and antioxidant properties [48]. Several pyrazole analogues, such as arylcydohexyl pyrazoles (W02017060855A1), n-aryl pyrazoles (W02018109642) and biaryl pyrazoles (W02017060854) are already established NRF2 regulators. The antioxidant effect of pyrazoles has been linked to the activation of the NRF2/KEAP1 signaling pathway. Thus, pyrazole (**3**) (Table 1) induces oxidative damage in NRF2 knockout mice but not in wild-type mice due to compensative enhancement of NRF2-regulated antioxidant capacity. Even when ROS is induced by cytochrome P4502E1 (CYP2E1/2A5) in NRF2 wild-type mice, pyrazole helps to attenuate the oxidative stress via the upregulation of the expression levels of NRF2 and NRF2-regulated antioxidant enzymes including HO-1, GST and GCS, contrary to what is observed in NRF2 knockout mice [49]. In corroboration with the fact that pyrazole requires NRF2 for its anti-oxidative action, liver injury increased as marked by serum transaminases and histopathology when NRF2 knockout mice were treated with pyrazole (**3**), but not in the NRF2 wild-type mice [50]. Contrary to expectations, pyrazole treatment did not elevate CYP2E1 and CYP2A5 activities in the NRF2 knockout mice, but increased their activities in the NRF2 wild-type mice. This confirms the earlier report that pyrazole-induced hepatotoxicity in the NRF2 knockout mice is independent of CYPZE1/2A5 induction [49]. In summary, it could be right to conclude that pyrazole significantly activates NRF2 and upregulates the expression levels of its target antioxidant genes such as HO-1, GCS, GST, and many more via a mechanism that does not involve the induction of CYP2E1/2A5.

Furthermore, pyrazole derivative (**4**) (Table 1) induces the NRF2 signaling pathway and inhibits glycogen synthase kinase-3β (GSK3β) [51]. The ability of compound **4** to activate NRF2 is ascribed to the presence of 2,4-dihydropyrano [2,3-*c*]pyrazole core, which also acts as a GSK3β inhibitor. Interestingly, the introduction of a pyrazole moiety to the curcumin scaffold improves the NRF2 activity and antioxidant capacity of curcumin. Evidently, the curcumin pyrazole derivative (**5**) (Table 1) has been found to exhibit better neuroprotective effects than curcumin and edaravone due to the pyrazole moiety [52]. Compound **5** also attenuates sodium nitroprusside (SNP)-induced oxidative damage and apoptosis, inhibits SNP-induced morphological changes, and protects the mitochondrial membrane via NRF2 activation in PC12 cells. Summarily, compound **5** provides neuroprotection and enhances the antioxidant defense system through the nuclear translocation of NRF2.

#### 2.1.3. Imidazolidine/Imidazole Analogues

Imidazolidine and imidazole are saturated and unsaturated five-membered *N*-heterocyclic rings, respectively. They contain two nitrogen atoms at the -1 and -3 positions. They are components of essential natural products, DNA based structures, and drugs. Imidazolidines are prepared by the condensation of aldehydes and 1,2-diamines, while imidazoles are produced by the condensation of glyoxal, ammonia and formaldehyde [53]. In addition, the cyclization of amido-nitriles is considered a notable synthetic procedure for disubstituted imidazoles [54]. Other imidazole derivatives are produced by facile synthetic methods which have been reviewed by [55]. Imidazolidines and imidazoles exhibit a broad spectrum of biological properties including anti-inflammatory and antioxidant activities [56,57]. Interestingly, some imidazole analogues have been found to be potent inducers of the NRF2/KEAP1 signaling pathway due to the fact that these analogues target several KEAP1 amino acid residues of NRF2 [58]. They exert NRF2-mediated antioxidant and anti-inflammatory effects in several diseases by undergoing the Michael addition reaction with the thiols of KEAP1 cysteine residues [58]. The authors of [59] reported that a compound containing imidazolide, a conjugate base of *IH*-imidazole–CCDO-imidazolide (**6**) (Table 1) is 100 times more potent than DMF, a known NRF2 activator, in the activation of the NRF2 signaling pathway. Compound **6** is a synthetic oleanane triterpenoid containing an imidazole ring. It inhibits the production of nitric oxide and attenuates ROS generation in RAW264.7 cells; it also induces about 52 NRF2-target genes, including NQO1, and HO-1 via NRF2 activation. The treatment with **6** attenuates the production of pro-inflammatory cytokine/chemokine, tubular injury and improves renal histology in mice via NRF2 activation and upregulation of antioxidant gene expression [60]. In a similar study, the compound **6** administration decreased oxidative/nitrosative stress, pro-inflammatory responses, and attenuated hepatic, pulmonary and renal damage in mice via NRF2 activation [61]. This type of NRF2 activation has also been linked to the amelioration of cardiac dysfunction and emphysema induced by cigarette smoke [62]. Furthermore, an imidazole analogue olmesartan (**7**) (Table 1) has been of therapeutic importance in hypertension. Although it contains another *N*-based heterocycle known as tetrazole, the imidazole ring is said to contribute majorly to the pharmacological properties of olmesartan (**7**), and its synthesis begins with an imidazole–dicarbonitrile scaffold [63]. Compound **7** exhibits significant antioxidant and anti-inflammatory activities. It inhibits oxidative stress in the daunorubicin (DNR)-induced nephrotoxicity in rats via the activation of the NRF2 signaling pathway and upregulation of the renal expression levels of GPX, Bcl–xL and PPAR-γ. By this activation process, it reduces oxidative stress and angiotensin II which are key to DNR-induced nephrotoxicity [64].

#### 2.1.4. Triazoles

Triazoles are unsaturated five-membered N-heterocyclic rings containing three nitrogen atoms. The large number of nitrogen atoms makes them chemically reactive and biologically important. They are commonly prepared via copper catalyzed cycloaddition reactions using calcium carbide as a source of acetylene [65]. Since the inception of click chemistry, Cu(1)-catalyzed azide-alkyne cycloaddition (CuAAC) has been used as a unique synthetic method for triazoles [66]. The synthesis of triazoles have been reviewed by [67]. Triazoles possess significant anti-inflammatory and antioxidant activities, and thus they have been extensively studied in neurodegenerative diseases [68,69]. The triazole derivatives (**8** and **9**) (Table 1) bearing 1,4-diaryl-1,2,3-triazole scaffolds significantly activate the NRF2 signaling pathway by inhibiting the KEAP1/NRF2 protein–protein interaction [70]. They also upregulate the expression levels of NRF2 dependent genes, including HO-1 and NQO1. 1,2,4-Triazole derivative (**10**) (Table 1) exerts a therapeutic effect in cerebral ischemic injury. It eliminates ROS, restores mitochondrial transmembrane potential, and attenuates neurological deficits in middle cerebral artery occlusion in acute ischemic stroke via NRF2 activation and induction of its antioxidant proteins such as HO-1, NQO1 and GCLC [71]. In a similar report, [72] reiterated that the neuroprotective effect of 1,2,4-triazole derivative (**11**) (Table 1) in cerebral ischemic injury is initiated by the antioxidant response element (ARE) and antioxidant genes HO-1 and NQO1 via the activation of the NRF2–ARE signaling pathway. Another 1,2,4-triazole analogue (**12**) (Table 1) with good bioavailability reportedly exhibited significant neuroprotective action against ischemic brain injury [73]. This implies that 1,2,4-triazoles could be an effective therapy in the treatment of ischemia related cases. Taken together, five-membered *N*-heterocyclic rings are endowed with diverse pharmacological properties, which accounts for the attention they have received in research lately. Particularly, their antioxidant and anti-inflammatory effects in biological systems exerted via the activation of the NRF2 signaling pathway are of notable medicinal interest.

### 2.2. Six-Membered N-Heterocyclic Rings and NRF2 Activation

Six-membered *N*-heterocycles are ubiquitous in natural products and bioactive molecules. Owing to their vast pharmacological properties, they are structural units of widely accepted pharmaceuticals, especially psychopharmaceuticals. Six-membered heterocycles containing one or two nitrogen atoms are components of drugs such as buspirone, hydroxyzine, trifluoperazine, amoxapine, trazodone, and many more [74,75]. Several compounds containing six-membered *N*-heterocycles activate the NRF2/KEAP1 signaling pathway [76].

#### 2.2.1. Piperidines

Piperidine is a saturated six-membered N-heterocycle present in several natural alkaloids and pharmaceuticals. It is produced by the reaction of piperine with nitric acid and, industrially, by catalytic hydrogenation of pyridine. It can also be synthesized by the reaction of *N*-(*tert*-butylsulfinyl)-bromoimine with Grignard reagents [77]. Recent advances in the synthesis of piperidines have been reviewed by [78]. Piperidine exhibits antioxidant and anti-inflammatory activities and has been utilized as an essential scaffold in drug discovery [79]. A piperidine alkaloid (piperine) (**13**) (Table 1) protects neuronal cells against H_2_O_2_-induced ROS accumulation, apoptosis and oxidative damage via NRF2-dependent phase II antioxidant enzymes, especially HO-1 and NQO1 [76]. Compound **13** exerts a significant neuroprotective effect for tyrosine hydroxylase-immunopositive dopaminergic neurons and attenuates behavioral deficits in 1-methyl-4-phenyl-1,2,3,6-tetrahydropyridine (MPTP)-induced Parkinson’s disease through the activation of the NRF2/KEAP1 signaling pathway. The authors of [80] reported that the cinnamyl piperidine analogue (**14**) (Table 1) inhibits neddylation, migration and increases apoptosis of gastric cancer cells via a process partly mediated by the NRF2–KEAP1 signaling pathway.

#### 2.2.2. Pyridine Analogues

Pyridine is an unsaturated six-membered *N*-heterocyclic ring with one nitrogen atom. It is commonly found in pharmaceuticals and vitamins. It can be produced industrially by the reaction of acrolein and acetaldehyde or through the biosynthesis of nicotinic acid [81,82]. In addition, Kröhnke pyridine synthesis has been a notable method which involves the reaction of α-pyridinium methyl ketone salts with α,β-unsaturated carbonyl compounds to produce pyridines [83]. Other methods of synthesizing pyridine have been reviewed by [84]. Pyridines possess antioxidant and anti-inflammatory properties [85,86]. Pyridine alkaloid (**15**) (Table 1) obtained from *Fusarium lateritium* enhances the expression of NRF2 and its target genes HO-1, and NQO1, thereby attenuating oxidative stress and apoptosis in glutamate-treated hippocampal HT22 cells [87]. This implies that the significant neuroprotective effects of pyridine can be attributed to its ability to activate the NRF2 signaling pathway. Pyridine derivative (**16**) (Table 1) also protects dopaminergic neurons from MPTP-induced oxidative stress; it suppresses the generation of proinflammatory enzymes and cytokines via the activation of NRF2 and upregulation of the *m*RNA levels of HO-1, SOD1, GCLM and GCLC, the NRF2-dependent antioxidant enzymes [88]. Through NRF2 activation, compound **16** restores the Parkinson’s disease-related motor dysfunctions in PD mice.

#### 2.2.3. Pyrimidine and Pyrazine Analogues

Pyrimidine is an aromatic six-membered *N*-heterocyclic ring with two nitrogen atoms at 1- and 3-positions of the ring. It is present in natural molecules such as alloxan, thymine, nucleotide cytosine and thiamine, as well as synthetic compounds such as barbiturates. Pyrimidines are produced via biosynthesis in the cytoplasm and chemically by the reaction of aryl ketones and anilines [89]. The cyclization reaction of ketones with nitriles under base has been found to be an economical synthetic procedure for pyrimidines [90]. Synthetic methods for pyrimidines have been reviewed by [91]. Pyrimidines are good antioxidants and anti-inflammatory agents [92,93]. Pyrazolo[3,4-*d*] pyrimidine derivatives exert therapeutic effects in neurodegenerative diseases. According to [94], these pyrimidine analogues activate the NRF2 signaling pathway. A pyrimidine analogue **17** (Table 1) has been found to elevate the *m*RNA and protein levels of NRF2-target antioxidant enzymes such as HO-1, NQO1, GCLM and GCLC in BV-2 cells. Through NRF2 activation, it exerts anti-inflammatory, antioxidant and neuroprotective effects. In addition to the upregulation of HO-1 via the activation of NRF2/HO-1 signaling, compound **17** also activates AMPK/HO-1 signaling and through these processes, it effects neuroprotection of nigral neurons in Parkinson’s disease [94]. In a similar development, Lee and co-workers [95] further corroborated that pyrazolo[3,4-*d*]pyrimidine (**18**) (Table 1) protects nigral dopaminergic neurons and inhibits the dopamine deficiency-related motor deficits via NRF2 activation and upregulation of HO-1, NQO1, GCLM and GCLC. Another Pyrazolo[3,4-*d*]pyrimidine derivative (**19**) (Table 1) ameliorates hepatic ischemia reperfusion injury in mice by inhibiting p21-activated kinase 4 (PAK4) due to its ability to stabilize NRF2 and enhance antioxidant capacity in mice [96].

Pyrazine which belongs to the same diazine class as pyrimidine has two nitrogen atoms in the 1- and 4-positions of the ring. Tetramethyl pyrazine (**20**) (Table 1) exhibits a significant antioxidant and anti-apoptotic activity in MPTP-induced Parkinson’s disease in mice via the upregulation of the expression levels of NRF2, GCLC, Bax and Bcl-2 [97].

#### 2.2.4. Triazines

Triazine is an unsaturated six-membered *N*-heterocyclic ring with three nitrogen atoms. They are commonly produced through Bamberger triazine synthesis which involves an aryl diazonium salt intermediate [98]. One-Pot synthesis through controlled cross-cyclotrimerization of nitriles is another efficient method for triazine preparation [99]. Other synthetic methods for the preparation of triazines have been reviewed by [100]. Triazines exhibit antioxidant and anti-inflammatory activities [101,102]. Triazines also exert neuroprotective effects in neurodegenerative diseases. Triazine analogues (**21** and **22**) (Table 1) maintain redox homeostasis, improve cell survival and enhance the overall antioxidant responses in organisms via the activation of NRF2 and upregulation of GPx1, GCS, SOD and CAT in neuronal cells [103]. Similarly, 1,2,4-triazine (**23**) (Table 1) inhibits H_2_O_2_-induced cell death, and exerts a neuroprotective effect in neuron-like PC12 cells via the activation of NRF2 and induction of GCS, HO-1 and GPX [104].

**Table 1:** Five- and six-membered nitrogen heterocyclic compounds and NRF2-inducing activities.

S/N	Molecule/Structure	Effective concentration(s)	NRF2 Target Genes	Disease of Interest	Study Model	Biological Activity of Interest	Reference(s)
1	**Pyrrolidine core** 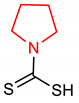 Pyrrolidine-1-carbodithioic acid	20 mg/kg	HO-1, NQO1, GCLM, GCLC	AD, Oxidative stress	Mice, Astrocytes	Antioxidant	[36]
		100 mg/kg		Infertility	Rats	Antioxidant, Anti-inflammatory	[37]
		50 mg/kg	GPx1, GPx4	Inflammation bowel disease (IBD)	Mice	Antioxidant, Anti-inflammatory	[38]
		100 µM	NQO1, GCLM	Oxidative stress	HepG2 Cells	GCL induction, NRF2 localization	[39]
		1–10 µM	HO-1, NQO1, GCLM, GCLC	AD, Aβ toxicity	Mice	Antioxidant, neurogenesis	[40]
2	**Pyrroline core** 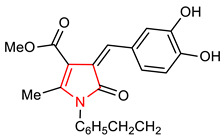 (*Z*)-Methyl-4-(3,4-dihydroxybenzylidene)-2-methyl-5-oxo-1-phenethyl-4,5-dihydro-1H-pyrrolin-3-carboxylate.	1 µM	HO-1, NQO1	Neurodegenerative diseases	SH-SY5Y Cells	Antioxidant	[41]
3	**Pyrazole core** 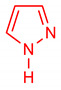 1*H*-Pyrazole	150 mg/kg	HO-1, GST	Liver injury, Oxidative stress	Mice	Antioxidant	[49]
		150 mg/kg	HO-1	Oxidative stress	Mice	Antioxidant	[50]
4	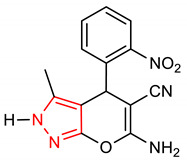 6-amino-3-methyl-4-(2-nitrophenyl) -2,4-dihydropyrano[2,3-c]pyrazole-5-carbonitrile	0.3–30 µM	HO-1, NQO1	AD, Oxidative stress	AREc32 Cells	Antioxidant, Anti-inflammatory	[51]
5	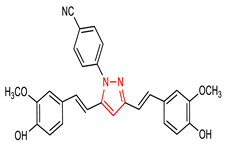 4-(3,5-bis((E)-4-hydroxy-3-methoxystyryl)-1H-pyrazol-1-yl)benzonitrile	1.25–5µM	GPx	Oxidative stress	PC12 Cells	Antioxidant	[52]
6	**Imidazole core** 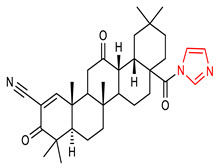 (4aR,6aS,12aS,12bS,14bR)-8a-(1H-imidazole-1-carbonyl)-4,4,6a,11,11,14b-hexamethyl-3,13-dioxo-3,4,4a,5,6,6a,6b,7,8,8a,9,10,11,12,12a,12b,13,14,14a,14b-icosahydropicene-2-carbonitrile	50–200 mg/kg	HO-1, NQO1	Lung cancer	Mice, RAW 264.7 Cells	Antioxidant, Anti-inflammatory	[59]
		30 µmol/kg	HO-1, NQO1, GCLC	Acute Kidney Injury	Mice	Antioxidant, Anti-inflammatory	[60]
		2 mg/kg	HO-1, NQO1, GCLC	Intestinal ischemia/reperfusion	Mice	Antioxidant, Anti-inflammatory	[61]
7	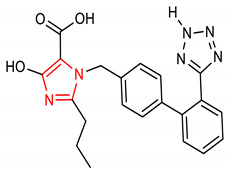 1-((2’-(2H-tetrazol-5-yl)-[1,1’-biphenyl]-4-yl)methyl)-4-hydroxy-2-propyl-1H-imidazole-5-carboxylic acid	10 mg/kg	GPx	Chronic nephrotoxicity	Rats	Antioxidant, Anti-inflammatory	[64]
8	**Triazole core** 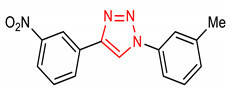 4-(3-nitrophenyl)-1-(m-tolyl)-1H-1,2,3-triazole	10 µM	HO-1, NQO1	Oxidative stress	HEK293 Cells, FP and NQO1 Assay	Antioxidant	[70]
9	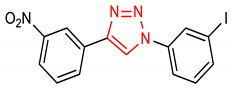 1-(3-iodophenyl)-4-(3-nitrophenyl)-1H-1,2,3-triazole	10 µM	HO-1, NQO1	Oxidative stress	HEK293 Cells, FP and NQO1 Assay	Antioxidant	[70]
10	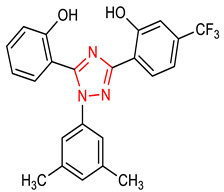 2-(1-(3,5-dimethylphenyl)-5-(2-hydroxyphenyl)-1H-1,2,4-triazol-3-yl)-5-(trifluoromethyl)phenol	<400 mg/kg	HO-1, NQO1	Ischemia stroke	Rats	Antioxidants	[72]
11	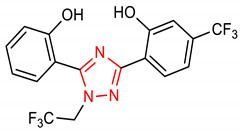 2-(5-(2-hydroxyphenyl)-1-(2,2,2-trifluoroethyl)-1H-1,2,4-triazol-3-yl)-5-(trifluoromethyl)phenol	<1000 mg/kg	HO-1, NQO1	Cerebral ischemic injury	Rats	Antioxidants	[71]
12	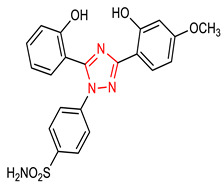 4-(3-(2-hydroxy-4-methoxyphenyl)-5-(2-hydroxyphenyl)-1H-1,2,4-triazol-1-yl)benzenesulfonamide	2.5–10 µM	GPx, SOD	Ischemic stroke	PC12 Cells	Antioxidant	[73]
13	**Piperidine core** 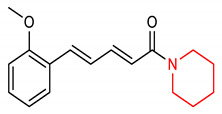 (2E,4E)-5-(2-methoxyphenyl)-1-(piperidin-1-yl)penta-2,4-dien-1-one	100 mg/kg	HO-1, NQO1	PD	PC12 Cells	Antioxiodant	[76]
14	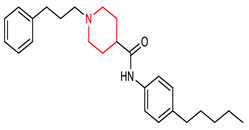 N-(4-pentylphenyl)-1-(3-phenylpropyl)piperidine-4-carboxamide		E1/E2/E2 enzymes	Gastric cancer	MIGC803 Cells	Anticancer	[80]
15	**Pyridine core** 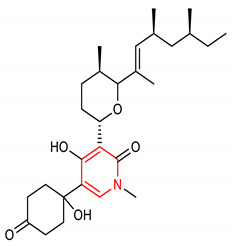 (+)-4,6-Anhydrooxysporidinone	2.5 and 5 µM	HO-1	Oxidative stress, apoptosis	HT22 cells	Antioxidant	[87]
16	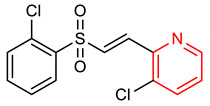 (E)-3-chloro-2-(2-((2-chlorophenyl)sulfonyl)vinyl)pyridine	30 mg/kg	HO-1, GCLC, GCLM, SOD-1	PD	Mice	Antioxidant, anti-inflammatory	[88]
17	**Pyrimidine core** 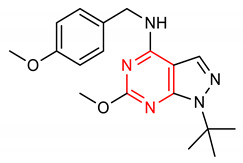 1-(tert-butyl)-6-methoxy-N-(4-methoxybenzyl)-1H-pyrazolo[3,4-d]pyrimidin-4-amine	30 mg/kg	HO-1, NQO1, GCLM,	PD	Mice	Antioxidant, anti-inflammatory	[94]
18	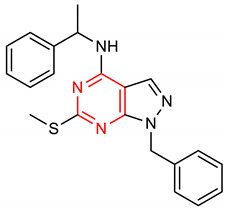 1-benzyl-6-(methylthio)-N-(1-phenylethyl)-1H-pyrazolo[3,4-d]pyrimidin-4-amine	2000 mg/kg	HO-1, NQO1, GCLM, GCLC	PD	Mice	Antioxidant, anti-inflammatory	[95]
19	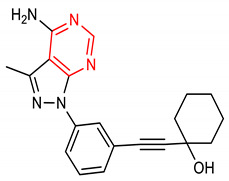 1-((3-(4-amino-3-methyl-1H-pyrazolo[3,4-d]pyrimidin-1-yl)phenyl)ethynyl)cyclohexanol	20 µM	HO-1, NQO1	Ischemia reperfusion injury	Mice	Antioxidant, anti-inflammatory	[96]
20	**Pyrazine core** 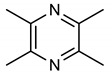 tetramethyl pyrazine	20 mg/kg	GCLC	PD	Mice	Antioxidant	[97]
21	**Triazine core** 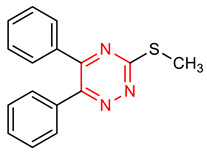 3-(methylthio)-5,6-diphenyl-1,2,4-triazine	10 µM	HO-1, GPx1	AD	PC12 Cells	Antioxidant	[103]
22	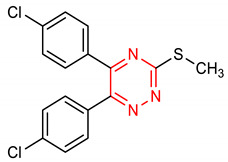 5,6-bis(4-chlorophenyl)-3-(methylthio)-1,2,4-triazine	10 µM	HO-1, GPx1	AD	PC12 Cells	Antioxidant	[103]
23	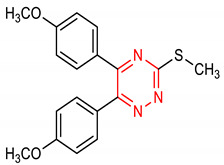 5,6-bis(4-methoxyphenyl)-3-(methylthio)-1,2,4-triazine	5–20 µ	HO-1, GPx1	AD	PC12 Cells	Antioxidant, anti-inflammatory	[104]

### 2.3. Fused/Condensed Nitrogen Heterocyclic Compounds

Fused nitrogen heterocycles contain at least one nitrogen heterocyclic ring fused with either a heterocyclic or carbocyclic ring. They have been extensively studied [105,106,107,108]. They are found in a wide range of bioactive natural products and synthetic compounds. Thus, almost a third of the best-selling therapeutics contain at least one fused heterocyclic compound, and the majority are nitrogen-based [1]. Apart from natural occurrence, several fused *N*-heterocycles are obtained via facile and effective synthetic processes [109]. Most of them exhibit antioxidant and anti-inflammatory activities [110].

#### 2.3.1. Indoles

Indole is an aromatic fused *N*-heterocyclic containing a benzene and pyrrole ring. They can be produced by certain bacteria or chemically by the catalytic reaction of aniline with ethylene glycol [111]. They can also be synthesized by acid–metal cyclization of aldehydes [112]. The synthesis of indoles has been reviewed by [113]. Indole-3-carbinol (I3C) (**24**) (Table 2), abundantly found in crucifers, regulates the NRF2 signaling pathway and exerts chemopreventive effects. The compound **24** induces ARE-luciferase activity and NRF2-mediated genes, and suppresses the incidence of palpable tumors and genitourinary weight [114]. It inhibits prostate cancer in transgenic adenocarcinomas of mouse prostate (TRAMP) mice via NRF2 activation. Although compound **24** is effective in activating the NRF2 signaling pathway, available data indicate that its dimerization to 3,3-iindolylmethane (**25**) (Table 2) results in improved NRF2-inducing activity. In a comparative study of their potential NRF2-inducing activity in murine fibroblasts (NIH3T3), compound **25** was found to induce the transactivation of NRF2 and upregulation of NQO1, γGCS and HO-1 in contrast to its precursor (**24**) [115]. In another development, the compound **25** suppresses DNMT expression, reverses the CpG methylation status of NRF2, upregulates the expression of NQO1 in vitro, and reduces tumorigenesis and metastasis in TRAMP mice via the activation of the NRF2 pathway which accounts for its chemopreventive actions in prostate cancer [116]. Prenylated indole alkaloid (**26**) (Table 2) also exerts neuroprotection against oxidative stress in SH-SY5Y cells via the nuclear translocation of NRF2 and the induction of NQO1 and HO-1. It activates NRF2 signaling by binding non-covalently with KEAP1, resulting in the reduction of ROS accumulation and the enhancement of the GSH level [117]. An indole analogue bearing a lactic acid moiety (**27**) (Table 2) attenuates inflammation and protects intestinal epithelial cells via the activation of NRF2 and aryl hydrogen receptor pathways [118]. Furthermore, an indole derivative (**28)** (Table 2) reduces ROS levels and improves neuronal viability in Parkinson’s disease via NRF2 activation [119]. It is important to note that several indole derivatives are non-covalent KEAP1-NRF2 protein–protein interaction (PPI) inhibitors. Through this mechanism, indole derivatives **29** and **30** (Table 2) increase the expression level of NQO1 and outperform *tert*-butylhydroquinone (*t*BHQ), a known NRF2 activator [120,121]. Some indole derivatives also regulate the induction of SOD2 via NRF2 expression in the mouse brain [122].

#### 2.3.2. Quinazolines

Quinazoline is an aromatic fused *N*-heterocyclic compound containing a benzene and pyrimidine ring. They are biologically active, and are components of several pharmaceuticals, including notable drugs [123]. They can be produced by reacting anthranilic acid and formamide in a process known as Niementowski’s synthesis [124]. The reaction of aromatic aldehydes with aminobenzimidazole and dimedone using sulfonic acid functionalized nano-porous silica has become a more convenient synthetic method for quinazolines [125]. Various methods used in the synthesis of quinazolines have been reviewed by [126]. Quinazolines possess diverse biological properties, including antioxidant activities [127,128,129]. Several quinazoline analogues of medicinal importance have been synthesized by the introduction of bioactive moieties to the stable quinazoline nucleus. The incorporation of nitrogen heterocycles at position 4- of the quinazoline ring has been found to enhance its cytoprotective activity including the activation of the NRF2 signaling pathway [130]. Quinozaline derivatives are highly potent inducers of the NRF2 target gene NQO1 [130]. The quinazolinone derivative (**31**) (Table 2) upregulates the expression levels of NRF2, HO-1 and NQO1, with a consequent downregulation of the expression of KEAP1, AhR and CYP1B1 [131]. This modulation of the AhR/CYP1B1/NRF2/KEAP1 signaling pathway by compound **31** accounts for its chemotherapeutic potency in the inhibition of liver carcinogenesis. Tryptanthrin, a natural quinazoline derivative (**32**) (Table 2) obtained from *Isatidis radix*, has been found to upregulate the expression levels of NRF2 and its target genes. Compound **32** also exhibits hepatoprotective effects against oxidative stress via the activation of the extracellular signal regulated kinase (ERK)/NRF2 signaling pathway in HepG2 cells [132]. On the contrary, indazolo[3,2-*b*]quinazolinones inhibit the NRF2/ARE signaling pathway; however, this opposing effect has been found therapeutic in hepatocellular carcinoma [133].

#### 2.3.3. Isoquinolines

Isoquinoline is an aromatic fused N-heterocycle made up of a benzene ring and a pyridine ring. They are isolated from natural alkaloids and produced chemically by Schlittler–Muller modification reaction [134,135]. They can also be prepared from benzaldehyde and amine via an acid-promoted synthesis [136]. Other synthetic methods for isoquinolines have been reviewed by [137]. Isoquinoline and its derivatives possess diverse biological properties, including antioxidant and anti-inflammatory activities [138,139]. Pyrazino[2,1-*a*]isoquinoline derivatives (**33** and **34**) (Table 2) are potent NRF2/ARE inducers [140,141]. Compounds **33** and **34** activate the NRF2/ARE signaling pathway and elevate NQO1 at the cellular level [140,141]. Diphenyl isoquinoline-I-amine derivative (**35**) (Table 2) exhibits anti-amnesic activity which has been linked to its ability to activate the NRF2/HO-1 signaling pathway. Through this activation, it attenuates oxidative stress and cholinergic dysfunction in the prefrontal cortex of mice exposed to scopolamine [142]. Furthermore, isoquinoline alkaloid (**36**) (Table 2) upregulates the expression of NRF2 transcription factor and its target genes such as HO-1, GPX, SOD, CAT and NQO1, which help in alleviating monosodium urate crystal-induced inflammation in rats [143].

**Table 2:** Fused Nitrogen heterocyclic compounds and NRF2-inducing activities.

S/N	Molecule/Structure	Effective Concentration(s)	NRF2 Target Genes	Disease of Interest	Study Model	Biological Activity of Interest	Reference(s)
24	**Indole core** 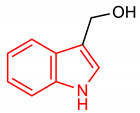 (1H-indol-3-yl)methanol	20mg/kg	NQO1	Prostate cancer	Mice	Antioxidant	[114]
25	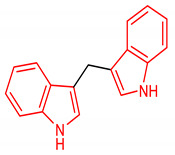 3,3′-diindolylmethane	25–100 µM	NQO1, HO-1	Oxidative stress	NIH3T3 Cells	Antioxidant	[115]
		5 µM	NQO1	Prostate cancer	TRAMP mice, C1 Cells	Antioxidant, Anticancer	[116]
26	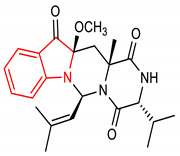 (3R,6S,12aR,13aR)-3-isopropyl-12a-methoxy-13a-methyl-6-(2-methylprop-1-en-1-yl)-2,3,13,13a-tetrahydro-1H-pyrazino[1’,2’:3,4]pyrimido[1,6-a]indole-1,4,12(6H,12aH)-trione	10–50 µM	NQO1	Oxidative stress	SH-SY5Y Cells	Antioxidant	[117]
27	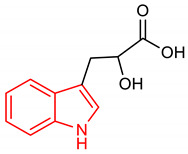 2-hydroxy-3-(1H-indol-3-yl)propanoic acid	0.1–10 mM	NQO1, SOD-2, GPX-2	Intestinal inflammation	Gut epithelial cells	Antioxidant, Anti-inflammatory	[118]
28	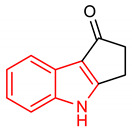 2,3-dihydrocyclopenta[b]indol-1(4H)-one	0.1 µM	NQO1	PD	SH-SY5Y	Antioxidant	[119]
29	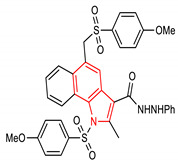 1-((4-methoxyphenyl)sulfonyl)-5-(((4-methoxyphenyl)sulfonyl)methyl)-2-methyl-N’-phenyl-1H-benzo[g]indole-3-carbohydrazide	4–100 µM	NQO1	Oxidative stress	MEF Cells, HepG2 Cells	Antioxidant	[120]
30	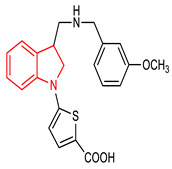 5-(3-(((3-methoxybenzyl)amino)methyl)indolin-1-yl)thiophene-2-carboxylic acid	5 µM	NQO1	Oxidative stress	HeLa Cells	Antioxidant	[121]
31	**Quinazoline core** 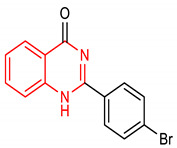 2-(4-bromophenyl)quinazolin-4(1H)-one	15, 30 mg/kg	NQO1, HO-1	Liver carcinogenesis	Rat	Antioxidant, Anticancer	[131]
32	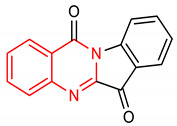 indolo[2,1-b]quinazoline-6,12-dione	1 µM	HO-1, GCLC	Oxidative stress	HepG2 Cells	Antioxidant	[132]
33	**Isoquinoline core** 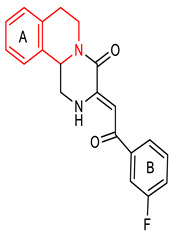 (Z)-3-(2-(3-fluorophenyl)-2-oxoethylidene)-2,3,6,7-tetrahydro-1H-pyrazino[2,1-a]isoquinolin-4(11bH)-one	10 µM	NQO1	Oxidative stress	HepG2-ARE-C8 Cells	Antioxidant	[141]
34	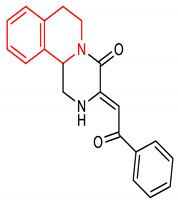 (Z)-3-(2-oxo-2-phenylethylidene)-2,3,6,7-tetrahydro-1H-pyrazino[2,1-a]isoquinolin-4(11bH)-one	10 µM	NQO1	Oxidative stress	HepG2-ARE-C8 Cells	Antioxidant	[141]
35	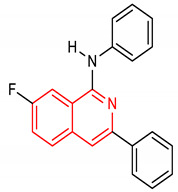 7-fluoro-1,3-diphenylisoquinolin-1-amine	10, 25 mg/kg	HO-1	Amnesia, Oxidative stress	Mice	Anti-amnesic, Antioxidant	[142]
36	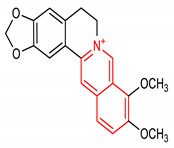 9,10-dimethoxy-5,6-dihydro-[1,3]dioxolo[4,5-g]isoquinolino[3,2-a]isoquinolin-7-ium	50 mg/kg	NQO1, HO-1, SOD-1, CAT, GPx	Gouty arthritis	Rats	Antioxidant, anti-inflammatory	[143]
37	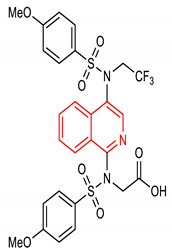 2-(4-methoxy-N-(4-(4-methoxy-N-(2,2,2-trifluoroethyl)phenylsulfonamido)isoquinolin-1-yl)phenylsulfonamido)acetic acid	15 µM	NQO1	Hepatic injury	U2OS Cells	Antioxidant, Hepatocellular protection	[144,145]

Several isoquinoline derivatives exert their NRF2-inducing activity by the inhibiting the KEAP1/NRF2 interaction. This is based on the fact that the pharmacological activation of NRF2 arises from the inhibition of the interaction of NRF2 with KEAP1 [144,145]. Thus, isoquinoline PRL-295 (**37**) (Table 2) increases KEAP1 thermostability in cell lysates and causes a disruption of its interaction with NRF2 in single live cells. This leads to the activation of NRF2 and enhanced hepatocellular protection. Oral administration of this isoquinoline analogue (**37**) in mice results in the induction of NQO1 in the liver, and a reduction of the plasma alanine aminotransferase and aspartate aminotransferase levels associated with acetaminophen-induced hepatic injury [145,145]. The modulation of NRF2 signaling pathway has been found therapeutic in hepatic diseases [146]. 

## 3. Pharmacological Profile of NRF2-Activating Nitrogen Heterocyclic Molecules

Several nitrogen heterocyclic derivatives exhibit good ligand and lipophilic efficiency, which influences their solubility, absorption, distribution and membrane permeability [147,148]. Compound **1** is a metal-chelating compound that permeates the blood brain barrier, making it valuable for CSN-related diseases [36]. It has a rapid absorption and intravenous LD_50_ of 282 mg/kg and 306 mg/kg in mice and rats, respectively. It possesses a toxicological profile that qualifies it as a drug candidate [149]. Pyrazole (**3**) exhibits good oral bioavailability, adsorption, distribution, metabolism, and excretion (ADME) and toxicological profile and drug-likeness [150].

In addition to higher NRF2-inducing activity and elevation of HO-1 and NQO1 *m*RNA levels, compounds **40** and **41** exhibit better metabolic stability and pharmacodynamics than compound **6** [151]. For instance, compound **6** is not as stable as compounds **40** and **41** in human plasma. Compounds **40** and **41** (12–15 µmol/kg) also exhibit a higher bioavailability of **6** (1.7 µmol/kg) in the mouse liver after six hours [151]. The metabolic and pharmacokinetic profiles of compound **7** have been reported by [152]. 1,4-diaryl-1,2,3-triazole derivatives (**8** and **9**) show strong binding interactions with Arg483, Arg415, Arg380, Ser602 and Asn382 amino acids of the Keap1 Kelch domain, making them act as Keap1-NRF2 PPI inhibitors [70]. The 1,2,4-triazole derivatives (**10**, **11** and **12**) have interesting pharmacokinetic properties. Compound **10** exhibits very suitable pharmacokinetic properties including low acute toxicity, a high plasma protein binding rate, and good hERG inhibition [72]. The pharmacokinetics and pharmacodynamics of derivatives of **13** have been evaluated by [153]. The cytotoxicity of pyridine derivative **16** shows 100% cell survival up to 10 µM, plasma and microsomal stability with about 97% and 66% of the intact remaining [107]. It also exhibits blood brain barrier permeability (P_e_: 65.16 × 10^−6^) which could be beneficial in CNS-related diseases [107]. Pyrimidine derivative **18** binds directly to KEAP1 with high affinity and dissociation constant (Kd) of 5.84 × 10^−10^ M and causes an alteration in the plasma resonance [108]. Pharmacokinetic studies indicate that **18** has good bioavailability and permeates the brain after intravenous and oral administration [108]. 1,2,4-Triazine derivatives (**21**, **22** and **23**) permeate the blood brain barrier [104]. Studies have revealed the bioavailability, metabolism and distribution of compounds **24** and **25**. While **24** is not detectable in plasma, **25** can be detected in plasma [154]. Compound **24** is highly unstable and rapidly absorbed to well-perfused tissues where it easily transforms to **25,** which is more stable and exerts anticancer actions [155]. Indole derivative **30** shows a strong binding interaction with amino acid residues of KEAP1 [121]. The pharmacokinetic and physicochemical properties of **30** have been reported by [121]. Isoquinoline derivative **33** exhibits unfavorable physicochemical properties such as poor membrane permeability (11.680 × 10^−6^ cm/s. pH 7.4) and water solubility (0.022 µg/µL, pH 7.4), probably due to the complexity of the ring systems and rigidity of the backbone [119]. However, these physicochemical properties were improved by the complete removal of benzene ring A (**48**).

## 4. Structure–Activity Relationship of NRF2-Activating Nitrogen Heterocyclic Molecules

**Figure 1:**
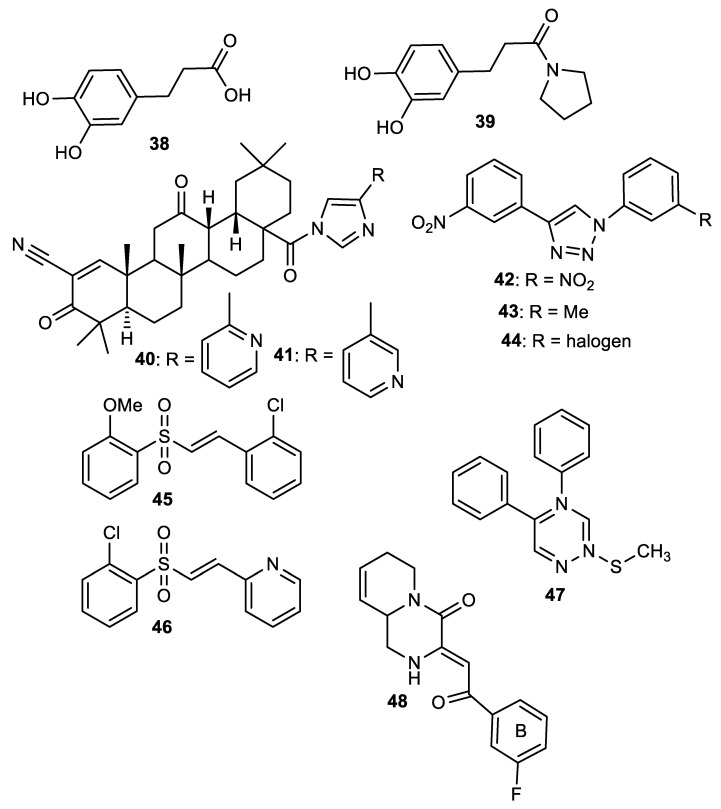
Structure–activity relationship (SAR) of NRF2-activating nitrogen heterocycle-containing molecules. The SAR assessment of molecules showed improved NRF2-inducing activity with the introduction of certain nitrogen heterocyclic compounds, as discussed in Section 4.

The SAR assessment of nitrogen heterocyclic molecules for improved NRF2-inducing activity is represented in Figure 1. The pyrrolidine moiety in compound **1** influences the antioxidant activity. The introduction of the pyrrolidine moiety to caffeic acid improves its antioxidant activity. The replacement of the OH of the COOH of caffeic acid with pyrrolidine increases its ability to attenuate lipid peroxidation and improve antioxidant capacity via the activation of Nrf2-dependent antioxidant enzyme HO-1 pathway and AKT pathway in heart [156]. The SAR studies of pyrazole derivatives indicate that the incorporation of the pyrazole core (**3**) increases their total antioxidant activity [157]. Although compound **6** activates the NRF2 signaling pathway and upregulates the expression levels of HO-1 and NQO1, the introduction of 2- and 3-pyridyl moieties to the imidazole produces better drug candidates **40** and **41**, respectively [151]. For 1,4-diaryl-1,2,3-triazoles (**8** and **9**), the insertion of a nitro group at the meta position of the 4-phenyl ring and a nitro (**42**), methyl (**43**) or halogen group (**44**) at the meta position of the 1-phenyl ring are the best conformations required for NRF2 cell-based activity [70]. For 1,2,4-triazole derivatives **10**, **11** and **12**, [72] reported that the introduction of alkyl groups at the 3-position of the 1,2,4-triazole moiety enhanced the NRF2-mediated neuroprotective effects. Notably, the 3,5-dimethyl substitution (**10**) confers the best NRF2-inducing activity and neuroprotection. For piperidine derivatives **13** and **14**, the introduction of *N,N*-dibutyl, *N,N*-dipropyl, *N,N*-*bis*trifluoromethyl or *p*-methyl to their piperidine scaffold enhances their pharmacological efficiency [158]. Compound **16** was designed based on SAR analysis, and it exhibits superlative NRF2-inducing activity. Among the drugs approved by the USA FDA, the pyridine moiety remains the second most commonly introduced aromatic N-heterocycle [159,160]. According to [107], the replacement of chlorobenzene with a pyridine ring and OMe with Cl- in vinyl sulfone (**45**: EC_50_ = 530 nM) improves its NRF2-inducing activity (**46**: EC_50_ = 0.618 µM). Furthermore, the insertion of 3-Cl into the pyridine ring of **46** confers the highest NRF2-inducing activity (**16**: EC_50_ = 0.026 µM).

A SAR evaluation of triazine derivatives (**21**, **22** and **23**) suggests that the introduction of aryl groups at 4-and 5-positions, and a thiolalkyl group at the 2-position of the triazine ring (**47**) could improve NRF2-mediated neuroprotective effects [103]. Structurally, compound **25** containing double indolyl groups outperforms its precursor (**24**) with one indolyl group as an NRF2 inducer [115]. The presence of double indolyl groups could be responsible for the increased NRF2-activating potency of compound **25**. The incorporation of a thiophene-carboxylic moiety improves the NRF2-inducing activity of indole derivatives [121]. The thiophene ring of compound **30** is involved in a strong interaction, which accounts for its ability to significantly induce NRF2-related antioxidant enzymes. The SAR of the isoquinoline derivative (**33**) has been studied. The benzene ring B in **33** has been identified as the main driver of its NRF2/ARE-inducing activity, and 3-F substitution of the benzene ring B (**33**) gives the best activity [141]. Removal of benzene ring A (**48**) results in comparable NRF2/ARE-inducing activity with **33** but improved physicochemical and drug-like properties.

## 5. Conclusions

Owing to their wide range of pharmacological activities, nitrogen heterocycles and analogues are essential candidate drugs for myriad of diseases, especially those in which oxidative stress and inflammation have been implicated. Interestingly, both natural and synthetic nitrogen heterocycles exert therapeutic effects in neurodegenerative diseases such as Alzheimer’s disease, Huntington’s disease, Parkinson’s disease, and many more [161,162,163]. This is due to the fact that most of these nitrogen heterocycles activate the NRF2 signaling pathway, which regulates oxidative stress and neuroinflammation, the key mediators in the development of neurodegenerative diseases. Through their NRF2-mediated antioxidant and anti-inflammatory effects, *N*-based heterocycles attenuate the gradual decline in neuronal functions associated with neurodegenerative diseases. Furthermore, the ability of these nitrogen heterocycles to elevate the expression levels of NRF2 target genes such as NQO1, HO-1, GCLM, GCLC, GPX, SOD and CAT represents an essential therapeutic strategy in a myriad of diseases. The available data indicate that about 95% of NRF2-activating nitrogen heterocycles induce the expression of NQO1 and HO-1, which are essential therapeutic molecular targets for several inflammation- and oxidative stress-mediated diseases. It is well established that while NQO1 catalyzes the reduction and detoxification of quinines and their analogues, HO-1 is involved in heme catabolism, and these processes exert anti-inflammatory and antioxidant effects in organisms. This implies that NRF2-activating nitrogen heterocycles will aid NQO1 and HO-1 targeted drug discovery for diseases in which oxidative stress and inflammation have been implicated. Taken together, the analyses of the NRF2-inducing activity of nitrogen heterocycles based on the size of the ring indicate that aziridines and azetidines which are three- and four-membered *N*-heterocycles, respectively, have not been explored yet. However, five-membered *N*-based heterocycles such as pyrrolidines, pyrroles, imidazoliding, imidazoles, triazoles and pyrazoles exert NRF2-mediated antioxidant and anti-inflammatory effects, which have been found therapeutic in diseases such as infertility, liver injury, inflammatory bowel diseases, lung cancer and neurodegenerative diseases, especially Alzheimer’s disease (Table 1). Furthermore, six-membered *N*-based heterocycles such as piperidines, pyridines, pyrimidines, pyrazines, triazines and their derivatives exhibit significant antioxidant and anti-inflammatory properties. They play essential therapeutic roles in Parkinson’s disease, gastric cancer, ischemia reperfusion injury and Alzheimer’s disease via NRF2 activation (Table 1). On the other hand, fused nitrogen heterocycles such as indoles, quinazolines and isoquinolines exhibit antioxidant and anti-inflammatory activities, and exert NRF2-mediated therapeutic effects in prostate cancer, intestinal inflammation, liver carcinogenesis, amnesia, gouty arthritis and Parkinson’s disease (Table 2). Obviously, higher membered rings such as azepine (seven-membered), azocines (eight–membered) and azonines (nine-membered) have not been explored. In the same vein, higher nitrogen containing heterocycles such as tetrazoles and pentazoles have not been subjected to NRF2-inducing activity evaluation. However, it is important to explore them because if nitrogen heterocycles were to activate NRF2 in direct proportion to their size and number of nitrogen atoms, then higher membered rings and higher nitrogen-containing heterocycles would be privileged molecules. In summary, based on NRF2-mediated activities, pharmacological profile and SAR evaluation, nitrogen heterocycles and their analogues represent good candidates for further development for inflammation and oxidative stress-mediated diseases, especially neurodegenerative diseases.

## Data Availability

Not applicable.
